# Man as an Object of Zoology

**Published:** 1887-11

**Authors:** S. W. Stiles

**Affiliations:** Georgia


### Southern Medical Record.

A MONTHLY JOURNAL OF PRACTICAL MEDICINE.

☞All Communications must be addressed to R. C. WORD, M. D., Managing Editor.

"*Quicquid Præcipies Esto Brevis.*"

VOL. XVII. ATLANTA, GA., NOVEMBER, 1887. NO. 11.

### ORIGINAL AND SELECTED ARTICLES.

### MAN AS AN OBJECT OF ZOOLOGY.

BY S. W. STILES, M.D., GA.

[*Continued from page 401.*]

Although it is a common and very just observation that two individuals are hardly to be met with possessing exactly the same features, and although this variety, according with what we observe throughout all nature, is a simple and effectual provision for very important ends; yet, there is generally a certain cast of countenance common to the particular races of men, and often to the inhabitants of particular countries. The five following varieties are established by Blumenbach, after a careful observation of the various races themselves in situations where commerce attracts them from all parts of the globe, as at London and Amsterdam, and drawings of the same:

1. Oval and straight face with the different parts moderately distinct from each other; high and expanded forehead; nose narrow and slightly aquiline, no prominence of the cheek bones, small mouth, lip is slightly turned out, particularly the lower one; a full and rounded chin. This is the kind of a countenance which accords most with our ideas of beauty.

2. Broad and flattened face with the parts slightly distinguished, and as it were running together, the space between the eyes flat and very broad, flat nose, rounded projecting cheeks, narrow and linear aperture of the eyelids, extending towards the temples, the internal angle of the eye depressed towards the nose, and the superior eyelid continued at that part into the inferior by a rounded sweep; chin slightly prominent. This is the face of the Mongolian tribes, commonly called in English the Tartar face.

3. Face broad, but not flat, and depressed with prominent cheek bones, and the parts, when viewed in profile, as it were more deeply carved out, short forehead, eyes deeply set, nose flattish, but prominent. Such is the countenance of most Americans.

4. Narrow face, projecting towards its lower part, narrow, slanting, and arched forehead; eyes prominent (a fleur de tete); a thick nose confused on either side with the projecting cheeks (nez epate); the lips, particularly the upper one, very thick; the jaws prominent, and the chin retracted. This is the countenance of the Negro, the Guinea face.

5. The face not so narrow as the preceding, rather projecting downwards, with the different parts, in a side view, rising more freely and distinctly. The nose rather full and broad, and thicker towards its apex (bottled nosed). The mouth large. This is the face of the Malay race.

In features, as in color, the different races are connected to each other by the most gentle gradations, so that although any two extremes when contrasted appear strikingly different, they are joined by numerous intermediate and very slightly differing degrees; and no formation is exhibited so constantly in all the individuals of one race, as to admit of numerous exceptions.

We see, indeed, an astonishing difference when we place an ugly Negro against a specimen of the Grecian ideal model; but when we trace the intermediate gradations, the striking diversity vanishes. A Creole Ynerdon, born of parents from Congo, and brought from St. Domingo, had a countenance, of which no part, not even the nose, and rather strongly marked lips, were very striking, much less displeasing; the same features, with an European complexion, would certainly have been generally agreeable. The testimony of Le Maire, in his journeys through Senegal, and Gambia, is to the same effect, and there are Negresses, except in color, as handsome as European women. Vaillant says of the Caffre women, that setting aside the prejudice which operates against their color, many might be considered handsome even in an European country. The accurate Adanson confirms this statement in his description of the Senegambians. I cannot help smiling, says Molina, when I read in certain authors, and those too accounted diligent observers, that all Americans have one cast of countenance, and that when you have seen one, you have seen the whole. The difference between an inhabitant of Chili and a Peruvian, is not less than between an Italian and a German. The truth of this representation is most fully stated by Humboldt, whose accuracy and extensive opportunities entitle his observations to the most implicit deference. The same style of features exist no doubt in both Americas, but those Europeans who have sailed on the great rivers Orinoco and Amazon, and have had occasion to see a great number of tribes assembled under the monastical hierarchy in the missions, must have observed that the American race contains nations whose features differ as essentially one from another, as the numerous varieties of the race of Caucacus, the Circassians, the Moors, and Persians differ from each other. From our survey of the countenance we proceed by a natural and easy transition to a consideration of the long head. I venture to affirm that a blind man, if he knew the vast difference which exists between the face of a Calmuck and that of a Negro, would be able to distinguish their skulls by the mere sense of touch, nor could you persuade any person, however ignorant of the subject, that either of these belonged to a head similar to those from which the divine examples of the ancient Grecian sculpture were copied.

[TO BE CONTINUED.]

